# Emergent clusteroluminescence from nonemissive molecules

**DOI:** 10.1038/s41467-025-59212-4

**Published:** 2025-04-25

**Authors:** Jianyu Zhang, Zuping Xiong, Haoke Zhang, Ben Zhong Tang

**Affiliations:** 1 MOE Key Laboratory of Macromolecular Synthesis and Functionalization, Department of Polymer Science and Engineering, Zhejiang University, Hangzhou, 310058 China; 2 Zhejiang-Israel Joint Laboratory of Self-Assembling Functional Materials, ZJU-Hangzhou Global Scientific and Technological Innovation Center, Zhejiang University, Hangzhou, 311215 China; 3 State Key Laboratory of Transvascular Implantation Devices, Hangzhou, 310009 China; 4 Guangdong Basic Research Center of Excellence for Aggregate Science, School of Science and Engineering, Shenzhen Institute of Aggregate Science and Technology, The Chinese University of Hong Kong, Shenzhen, Guangdong 518172 China

**Keywords:** Organic molecules in materials science, Optical materials, Polymers

## Abstract

Once considered the exclusive property of conjugated molecules, efficient and visible-light luminescence from non-conjugated and nonemissive molecules in the clustered state, known as clusteroluminescence (CL), has attracted much attention recently due to its special photophysical behaviors and behind electronic interactions. This perspective discusses the development of the CL phenomenon, followed by the typical photophysical features, examples, mechanisms, and potential applications of CL materials, to provide a comprehensive picture of this emerging field. Starting with organic clusters, inorganic, metallic, and hybrid clusters with CL properties are also introduced, and the perspective shift from covalent interactions at the molecular level to non-covalent interactions at the aggregate level is invoked.

## Abstract (web summary)

Clusteroluminescence from non-conjugated and nonemissive molecules is an area of increasing interest. Here, the authors consider the development of the field, as well as potential future directions.

## Introduction

The interaction of light with matter promotes the development of numerous scientific theories and life-changing innovations^1,2^. Apart from the exploration of the natural light source, the development of artificial light sources has attracted much attention during the past several decades, especially for organic emitters with the advantages of lightweight, rich colors, and large-scale processability^3,4^. Systematic theories for organic optoelectronics were gradually established, which highlights the dependence of material functionalities on the fundamental properties of a single molecule. From this perspective, extended π/*n*-electron conjugation and related charge-transfer (CT) processes through covalent bonds among molecular skeletons seem necessary to achieve efficient luminescent properties of molecules, which have also been proven as effective and mature strategies for constructing organic emitters^5,6^. Therefore, less attention is paid to these non-conjugated and seemingly nonemissive molecules, and even macroscopic materials made from them.

Thinking outside of the box of covalent interactions at the molecular level, non-covalent interactions usually existing in the aggregate state also play crucial roles in physical and life sciences^7,8^. For traditionally conjugated systems, *H*- and *J*-aggregation have been proven to greatly affect their aggregate-level phenomena. However, due to the limited understanding and lack of aggregate models of non-conjugated molecules, the effects of non-covalent weak interactions on the electronic structure of aggregates and its related luminescent properties have rarely been reported^9^. Thus, it is still challenging but would be a perspective shift to illustrate the relationship between non-covalent interactions and luminescent behavior at clustered and higher aggregate levels, especially for these non-conjugated molecule-based materials. These non-covalent interactions of electrons are believed to supplement the covalent interaction-based optoelectronic theories, and also alter aggregate structures and macroscopic performance of light-emitting materials, providing a platform to understand some emergent phenomena observed in aggregates but disappeared at the molecular level^10,11^.

**Fig. 1:**
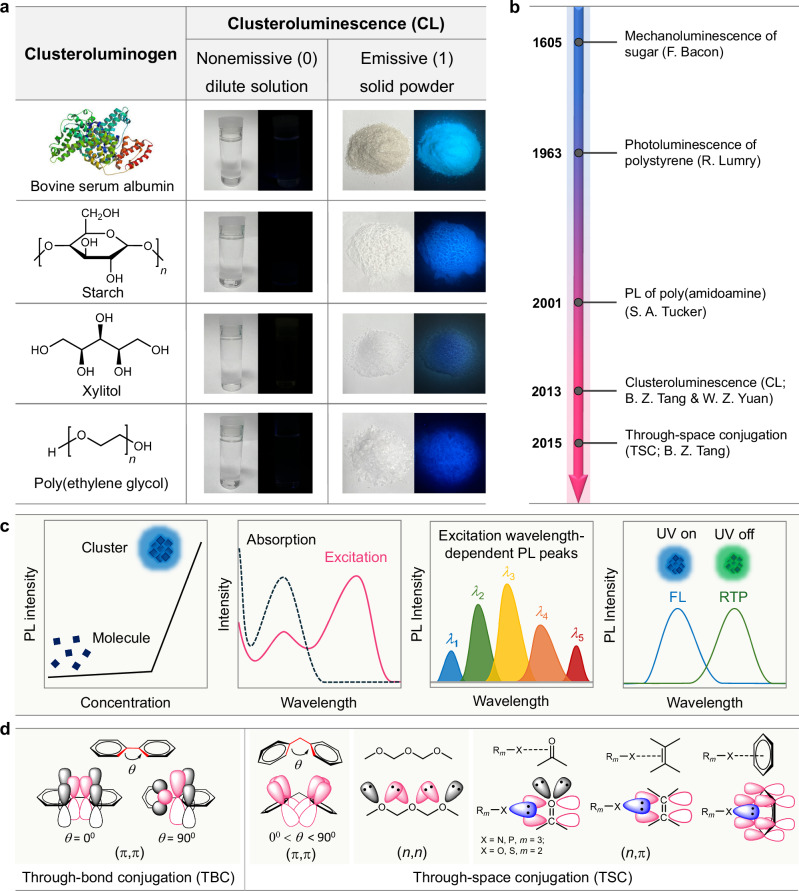
The phenomena and development of clusteroluminescence (CL) and typical features of clusteroluminogens. **a** The emergence of CL from nonemissive (macro)molecules (“0”) in the dilute solutions to emissive clusters (“1”) in the solid state. **b** The important milestones in the historical development of CL research. **c** The diagrammatic illustrations of photophysical characteristics of clusteroluminogens; PL = photoluminescence, FL = fluorescence, RTP = room-temperature phosphorescence. **d** Examples of (π,π), (*n*,*n*), and (*n*,π) electronic interactions (TBC and TSC) in the luminogenic systems.

In recent years, it has been found that some natural proteins, macromolecules, and even small molecules, such as bovine serum albumin, starch, and xylitol, could emit abnormal blue light in the solid state, despite being non-conjugated or completely free of π electrons (Fig. 1a)^12,13^. In fact, the earliest report of luminescence from non-conjugated materials can be traced back to the mechanoluminescence of solid sugar more than 400 years ago discovered by Bacon (Fig. 1b)^14^. Later, Lumry et al. reported the long-wavelength emission of isotactic polystyrene (PS) in 1963^15^, and Tucker et al. studied the luminescent behavior of poly(amidoamine) in 2001^16^. However, these isolated examples did not attract much attention due to their poor luminescent properties, unclear mechanism, and constraints of traditional optoelectronic theories. Besides, people were more likely to believe that their luminescence came from impurities inside materials. Thus, these non-conjugated materials and peculiar luminescence remain unsealed until recent years. In 2013, Tang and Yuan et al. revisited the unconventional luminescent behaviors of a series of polymers and small molecules^13,17^. Since these non-conjugated materials, in most cases, were totally nonemissive as isolated molecules in dilute solution but became emissive with extrinsic long-wavelength light in the clustered state (Fig. 1a), they coined this phenomenon clusteroluminescence (CL). In 2015, with a strong interest in CL, Tang et al. carefully studied the photophysical properties of tetrahydropyrimidines and proposed the through-space conjugation (TSC) as the working mechanism behind this phenomenon, opening the fast track for CL study^18,19^.

Compared to traditional conjugated compounds with involuted synthetic procedures, high costs, and solubility issues for fabrication, these non-conjugated materials, characterized by their simple structures, can be sourced from nature (e.g., polysaccharides, lignin, biomass) or synthesized using straightforward methods and basic starting materials. Hence, they exhibit numerous advantages, including high biocompatibility, renewability, low toxicity, and potential biological activity. Given their high theoretical value and broad practical prospects, a growing number of CL materials have been discovered and studied over the past decade, with a focus on their mechanisms, property modulation, and synthetic methods. Some emergent CL materials have achieved performance comparable to traditional conjugated luminescent materials with wide emissive colors (from blue to NIR region), high efficiency (up to 100%), and multiple applications^20,21^. CL not only constructs a system of luminescent materials based on non-conjugated structures but also facilitates a perspective shift from molecular science to aggregate science^22^.

Herein, in this perspective, we summarize the key features, examples, and applications of organic CL materials, with a particular emphasis on the fundamental mechanisms of TSC. Moreover, we aim to offer an insightful overview of the current state of CL and to invigorate further developments in this rapidly advancing field. Meanwhile, we expand the organic cluster to inorganic, metal, and hybrid clusters with CL properties to explore the close relationships between CL of different systems and weak through-space interactions at the aggregate level. Finally, arising from physical and life science, we illustrate the perspective shift from the covalent-bond theory of single molecules to the non-covalent interactions of aggregates. It is believed that the advancement of CL can pave the way for a different type of photo-functional materials and establish a research field of aggregate science.

### Typical features and representative examples of CL

In terms of the luminescent phenomenon, CL is a special type of aggregation-induced emission (AIE) since they all show enhanced emission and restriction of molecular motions effect after aggregation. Besides, CL exhibits several typical features that are different from widely acknowledged AIE luminogens. Thus, to provide a general picture of CL, the typical features of luminogens with CL properties (CLgens) are first discussed and compared with two kinds of popular luminogens with aggregation-caused quenching (ACQgens) and AIE (AIEgens) effects (Table 1 and Fig. 1c-d)^23^. In terms of electronic interaction, ACQgens with planar conformation own covalently through-bond conjugation (TBC), which show intrinsic single-molecule emission in dilute solution and suffer the quenching effect in the aggregate state^24,25^. With many rotors and twisted conformation, AIEgens achieve luminescence via both or one of the TBC and TSC channels. Although multiple intermolecular interactions exist in the aggregate state, they are too weak to alter the electronic structure of most AIEgens and mainly restrict intramolecular motions to achieve enhanced emission from single-emissive species^26^.

**Table 1:** The comparison of luminogens showing aggregation-caused quenching (ACQgens), aggregation-induced emission (AIEgens), and clusteroluminescence (CLgens) effects

Feature	ACQgens	AIEgens	CLgens
Electronic interaction	TBC	TBC/TSC	TSC
Emission upon aggregation	Weakened or quenched	Intensified	Activated
Emitting species	Single molecule (monomer)	Monomer	Mono-/multimer (cluster)
Absorption & excitation spectra	Identical	Identical	Different
Excitation wavelength dependence	Independent	Independent	Dependent
Emission spectrum	Single-peaked	Single-peaked	Multi-peaked
RTP	Seldomly	Occasionally	Commonly

In comparison, CLgens with non-conjugated structures only rely on the TSC in the clustered state, showing some distinctive features that differ from TBC-based ACQgens. First, the active molecular motions of flexible skeletons could be restricted when CLgens form clusters, endowing them to form stable clusters as emissive species and activating emission (Fig. 1c)^27^. It is worth noting that the emergent CL always displays a longer wavelength than the intrinsic emission from isolated units or single molecules. Second, the strength of the electronic TSC is much weaker than covalent TBC. Thus, the low-sensitivity absorption characterization can not capture the absorption signal of the long-wavelength transition with low oscillator strength, resulting in unmatched absorption and excitation spectra of CLgens^28^. Third, different from TBC, TSC is more sensitive to molecular conformation, the distance between atoms, and the orientation of electronic orbitals, indicating that intramolecular and intermolecular TSC in the clustered state can compose multiple emissive species simultaneously^29^. According to molecular photophysical theories, electron transition from excited state to ground state follows Kasha’s rule, that electron would be released radiatively from the lowest-excited state of each emissive species with a fixed energy gap^30^. As a result, ACQgens and AIEgens usually show excitation-wavelength-independent emission, while CLgens always exhibit excitation-wavelength-dependent CL with different emission wavelengths and quantum yields under different excitation wavelengths^31^. Fourth, apart from the non-conjugated structure, many CLgens are rich in electron-rich heteroatoms (e.g., O, N, S, and P), leading to various electronic interactions between *n* and *n*, π and π, and *n* and π electrons, referring to the (*n*,*n*), (π,π) and (*n*,π) TSC, respectively. These heteroatoms and hybrid transition features are beneficial for efficient intersystem crossing, prompting the commonly observed room-temperature phosphorescence (RTP) from CLgens^32–34^. In contrast, RTP is seldom noticed in pure hydrocarbon systems. In terms of the difference between TBC and TSC, although the strength of both conjugations is highly dependent on the twisted angle, a planar conformation is more beneficial for TBC while a twisted conformation is conducive to TSC (Fig. 1d). For example, in biphenyl, conjugation is completely broken when the two benzene rings become perpendicular. However, the TSC of diphenylmethane exhibits a high tolerance for conformational changes due to its multichannel conjugation feature. The above features represent the fundamental parameters for determining CLgens, which also suggest CLgens as a different type of luminescent material with more controllability and potential multifunctional properties.

**Fig. 2:**
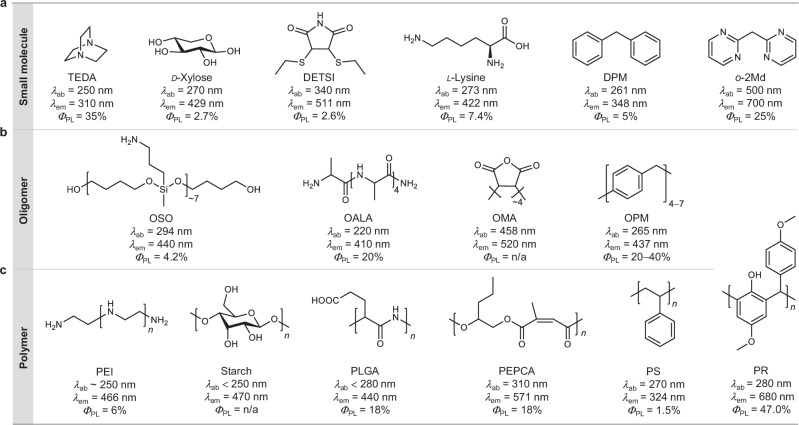
Typical examples of chemical structures of CLgens. **a** Small-molecule CLgens. **b** Oligomer-based CLgens. **c** Polymer-based CLgens. Abbreviations: *λ*_ab_ = absorption maximum, *λ*_em_ = emission maximum, *Φ*_PL_ = absolute photoluminescence quantum yield, TEDA = triethylenediamine, DETSI = 2,3-diethylthiosuccinimide, DPM = diphenylmethane, *o*-2Md = di*(o-*pyrimidinyl)methane, OSO = oligo(siloxane), OALA = oligo(*L*-alanine), OMA = oligo(maleic anhydride), OPM = oligo(phenylene methylene), PEI = polyethylenimine, PLGA = poly(*L*-glutamic acid), PEPCA = poly[(1,2-epoxypentane)-*alt*-(citraconic anhydride)], PS = polystyrene (*isotactic*), PR = phenolic resin.

Following some CLgens originating from natural systems, many artificial small molecules and polymeric materials were synthesized to clarify the underlying photophysical processes and pursue better properties. Here, three types of typical CLgens are listed with their absorption (*λ*_ab_) and emission maximum (*λ*_em_), and absolute photoluminescence quantum yield (*Φ*_PL_), including small molecules, oligomers, and polymers (Fig. 2). In general, their chemical structures are non-conjugated with heteroatoms, saturated alkanes or isolated aromatic rings. However, they can produce visible and even near-infrared (NIR) emissions in the aggregate state, which goes outside the limitations of traditional TBC-based theories. For small-molecule CLgens, triethylenediamine is a typical example without π electrons but can emit light at 310 nm (Fig. 2a)^35^. *D*-xylose is a representative of polysaccharides with multiple oxygen atoms and free of π electrons, which can emit blue light at 429 nm with a *Φ*_PL_ of 2.7% in the crystalline state^36^. The solid-state diethylthiosuccinimide (DETSI) with a non-conjugated structure shows emission at 511 nm due to (*n*,π*) transition, which is redshifted and more efficient than its derivative without the ethylthio unit^37^. Many natural and nonaromatic amino acids were also found to show intrinsic visible emission, as represented by *L*-lysine with sky-blue emission in the solid state^12^. Additionally, luminogens composed of isolated aromatic rings linked by alkyl chains, known as multiarylalkanes (MAAs), are another typical class of CLgens that exhibit visible-light emission. The most simple MAA is diphenylmethane (DPM) with two benzene rings connected by a methylene unit, which can form TSC of π electrons (also known as homoconjugation)^38,39^. As a result, it emits dark-purple fluorescence at 348 nm in the solid state, which is much longer than the intrinsic emission of benzene rings at 278 nm^40^. Introducing lone pairs into this system results in di(*o*-pyrimidinyl)methane (*o*-2Md) with largely redshifted emission at 700 nm and increased *Φ*_PL_ of 25%, which may be the smallest luminogen with NIR emission as present^41^.

Oligomers are more likely to form clusters than small molecules in poor solvents or concentrated solutions, showing brighter CL (Fig. 2b). Siloxane is a kind of oxygen-involved system without π electrons, which has been proven as a good candidate for CL materials^42^. For instance, oligo(siloxane) shows excitation/concentration-dependent luminescence and inherent blue luminescence from formed oxygen clusters at 440 nm under 365 nm UV lamp illumination^43^. Peptides with abundant amide groups also exhibit excellent CL properties, associated with TSC between amide groups triggered by multiple hydrogen bonds^44^. Oligo(*L*-alanine) is a typical example of oligomeric peptide with blue CL at 410 nm and a high *Φ*_PL_ of 20%^45^. The carbonyl group was proved to be an important building unit for CL due to intra- and inter-chain (*n*,π*) transition. Accordingly, oligo(maleic anhydride) (OMA) with carbonyl groups is an oligomeric representative whose solid sample shows CL at about 520 nm^46^. Interestingly, the non-conjugated DPM could be expanded to oligo(phenylene methylene)s (OPMs) with a different number of units. These OPMs exhibit almost the same emission wavelength of CL at 437 nm but different *Φ*_PL_ of 20−40% depending on the balance of skeleton flexibility and rigidity^47^. Additionally, increasing the number of repeating units can convert oligomers into polymers (Fig. 2c). In this category, polyethylenimine (PEI)^48^, starch^13^, and poly(*L*-glutamic acid) (PLGA)^49^ are polymeric representatives of pure *n*-electron system, polysaccharides, and polypeptides, respectively, which all show blue CL within 440−470 nm. Besides, polyesters are widely studied CLgens with good controllability of luminescent properties through main and side chains^15,21^. For example, PEPCA with through-space (*n*,π) interactions displays high-efficiency green CL at 571 nm^50^. By introducing electron-donating groups and isolated benzene rings, the well-developed engineering plastics of phenolic resins (PR) demonstrate obvious CL, reaching the NIR region at 680 nm with a high *Φ*_PL_ of 47%^51^. Although some of the above examples have shown excellent performance, the properties of most CLgens are still poor (e.g., blue-region wavelength and low efficiency), seriously hindering the development of their potential applications.

### Working mechanisms and manipulating strategies of CL

**Fig. 3:**
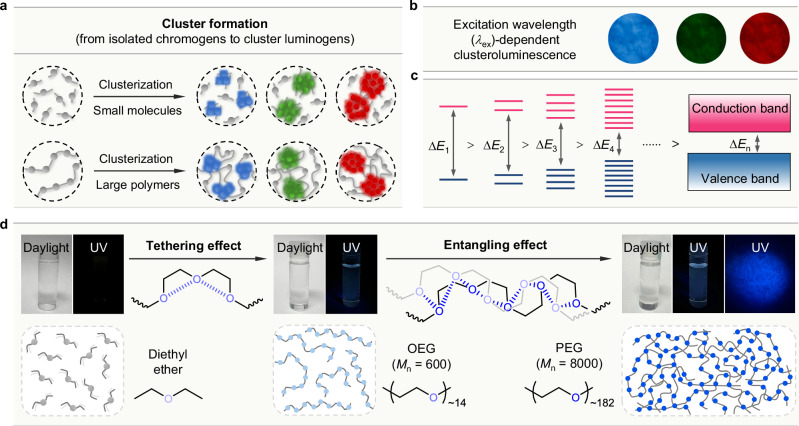
Electronic interactions and clusterization processes. **a** The diagrammatic illustrations of the cluster formation from isolated chromogens to cluster luminogens in molecular and polymeric systems. **b** The excitation wavelength-dependent CL of sodium alginate powders; photographs taken under the illumination of (from left to right) UV, blue, and green lights, respectively. **c** The increased through-space electronic interactions among the chromogenic units in the larger clusters lead to more energy-level splittings and smaller energy gaps (Δ*E*). **d** The polymer effect in the CL systems: the tethering effect in the low-molecular-weight oligo(ethylene glycol) (OEG) facilitates the cluster formation through the (*n*,*n*) TSC between the oxygen atoms in the short oligomer chain, while in the high-molecular-weight poly(ethylene glycol) (PEG), the synergistic entangling effect (between different polymer chains) and tethering effect (along same polymer chain) results in the formation of oxygenic CLgen networks. The photographs of pure diethyl ether (liquid), pure OEG (liquid), a concentrated PEG solution (100 mM in water), and PEG powders were taken under daylight and UV illumination. **b** is adapted with permission from ref. ^52^ Copyright (2018) American Chemical Society.

Mechanistic studies, which illustrate the structure-property relationship and regulation strategies of performance, play a crucial role in CL research. As discussed above, in most cases, CLgens are nonemissive as isolated chromogens but produce emergent CL in concentrated solutions or the solid state, suggesting the crucial process of clusterization for TSC (Fig. 3). For both small molecules and large polymers, they can form different cluster luminogens with different sizes through intermolecular interactions (e.g., electrostatic, dipole, and dispersion interaction) or chemical bonds (e.g., polymerization and coordination) (Fig. 3a). Usually, clusters with different sizes would produce different emission colors due to the various strength and degree of TSC, which results in the excitation-wavelength-dependent CL as observed in many systems. For example, the natural anionic polysaccharide of sodium alginate can emit blue, green, and red CL in the solid state under the illumination of UV, blue, and green lights, respectively (Fig. 3b)^52^. The working mechanism of CL is closely related to the energy-level splitting, similar to the band theory applied to inorganic emitters (Fig. 3c)^53^. In the clustered state, electron-rich units get close to each other, and the restricted molecular motions can stabilize excited-state geometries of formed clusters, promoting the formation of through-space electronic coupling and delocalization. With the increased number of delocalized electrons, the energy gap gradually narrows, potentially forming an electron band that ultimately leads to the observed long-wavelength emission^54^. Based on this mechanism, it is worth noting that CL could also be achieved from a few molecules in the isolated state or rigid matrix once their TSC could be stabilized and strengthened. In addition, due to the comparatively weak TSC and different mechanisms of CLgens compared to TBC-based systems, the traditional frontier molecular orbital theory fails to accurately describe the electronic structure and energy gap of these CLgens in most cases^55^.

The transformation from nonemissive diethyl ether to PEG with bright blue-light luminescence is a good example to demonstrate the working mechanism of cluster formation and CL (Fig. 3d). In the liquid state, the distance between oxygen atoms of two diethyl ether molecules is too far apart to enable strong interactions. Therefore, no emissive signal could be observed. Connecting several repeating units of diethyl ether via covalent bonds affords low-molecular-weight oligo(ethylene glycol) (OEG), which plays the tethering effect of facilitating intramolecular (*n*,*n*) TSC by bringing two oxygen atoms closer together with a shorter distance than the sum of their van der Waals radii^56^. Thus, the (*n*,*n*) TSC endows liquid-state OEG with blue CL, although the emission intensity is comparatively weak. For high-molecular-weight poly(ethylene glycol) (PEG), apart from the intramolecular tethering effect, the entangling effect between different polymer chains forms inter-chain O···O networks, which rigidify the PEG chains and enhance the strength of (*n*,*n*) TSC. The crystal structure of PEG also proves its networks with short O···O distances^57^. As a result, PEG produces stronger blue CL than liquid OEG in concentrated aqueous solution and the powder state^58^.

**Fig. 4:**
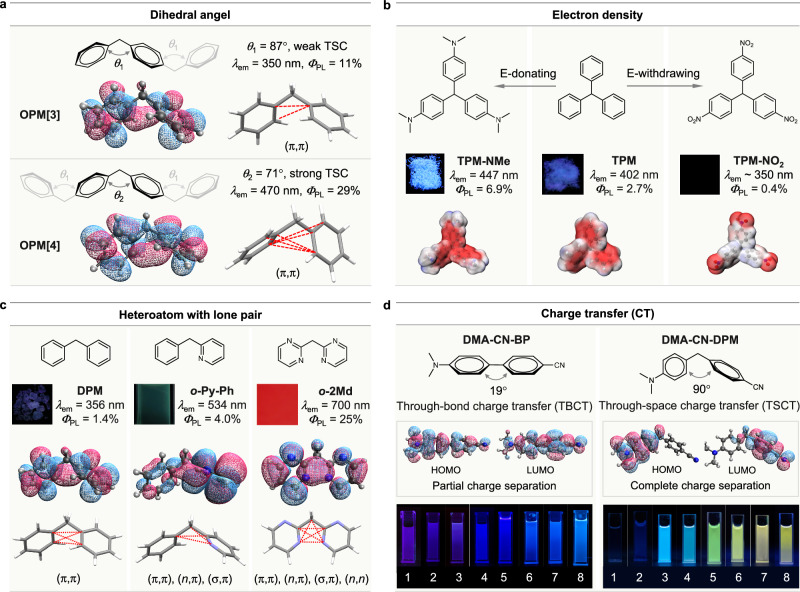
Factors affecting through-space conjugation (TSC) and CL process. **a** Dihedral angles between the phenyl rings (*θ*_1_ and *θ*_2_) in the oligo(phenylene methylene) (OPM[n], n = 3, 4) chain. The diphenylmethane (DPM) unit with appropriate dihedral angle favorites intramolecular (π,π) TSC leads to a red-shift in emission color and an increase in quantum yield (*Φ*_PL_). **b** The CL behaviors and electrostatic potential maps of triphenylmethane (TPM) and its derivatives carrying electron-donating (TPM-NMe) and electron-withdrawing (TPM-NO_2_) substituents (isovalue = 0.02, GaussView). **c** The CL behaviors, LUMO diagrams (isovalue = 0.05, IQmol), and intramolecular interactions of DPM and its derivatives containing heteroatoms; the modes of TSC [(π,π), (*n*,π), (σ,π), (*n*,*n*)] are increased with increasing the number of nitrogen atoms in the DPM derivatives. **d** The extent of charge separation (isovalue = 0.05, IQmol) and solvatochromic effect in the luminogens involving TBCT and TSCT processes: the emission color of the CLgen with complete charge separation (DMA-CN-DPM) bathochromically shifts to a larger extent with an increase in the solvent polarity (1 = hexane, 2 = toluene, 3 = tetrahydrofuran, 4 = chloroform, 5 = acetone, 6 = dimethylformamide, 7 = dimethyl sulfoxide, 8 = acetonitrile); concentration is 5 × 10^-4^ M. **b** is adapted with permission from ref. ^65^ Copyright (2021) American Chemical Society. **c** is adapted with permission from ref. ^41^ Copyright (2025) Cell Press. **d** is adapted with permission from ref. ^40^ Copyright (2024) Springer Nature.

Since the complex electronic structure and limited models of clusters, polymeric CLgens are not ideal systems for structure-property study. In contrast, small molecules, with their precise conformation and crystalline packing, are better suited for investigating the factors influencing TSC and the manipulation strategies for CL. Notably, many studies focus on multiarylmethanes, which can be considered small CLgens comprising two or three aryl units connected by a saturated methyl unit (Fig. 4)^59–63^. First, due to the shape and direction of *n*/π-electron orbitals, the conformation of CLgens is crucial for electronic overlap to achieve strong TSC^20^. For example, the oligomer OPM[4] with a small dihedral angle of 71^°^ displays a stronger TSC with multiple intramolecular *p*-orbital interactions between two isolated benzene rings (Fig. 4a). However, OPM[3] exhibits a comparatively large dihedral angle of 87^°^ and weak TSC. As a result, the CL wavelength of OPM[4] reaches 470 nm with a high *Φ*_PL_ of 29%, while the wavelength of OPM[3] is only located at 350 nm with a low *Φ*_PL_ of 11%^47^. In addition, by changing the connecting position of terphenyl from *p*-terphenyl to *o*-terphenyl, the dihedral angle between each benzene ring increases, which makes it change from an ACQgen to CLgen with the mechanism shift from TBC to TSC^64^. Second, based on traditional photophysical theory, the higher the electron density, the stronger the conjugation effect. Accordingly, the CL properties of triphenylmethane (TPM) and its derivatives with different substituents were studied and compared (Fig. 4b)^65^. It illustrates that electron-donating groups of TPM-NMe could enhance the electron density at the central position of the molecular skeleton (as shown by the electrostatic potential map with red color), which simultaneously stabilizes the excited-state geometry and strengthens TSC, resulting in redshifted wavelength (from 420 nm to 447 nm) and increased efficiency (from 2.7% to 6.9%) compared to TPM. In contrast, electron-withdrawing groups of TPM-NO_2_ would decrease the electron density for TSC and quench CL.

Adjacent heteroatoms can introduce *n*-electron-involved interactions and facilitate cluster formation for stronger TSC^66^. Thus, the third strategy for regulating CL is to construct TSC involving lone pairs between heteroatoms, as explained by the different CL properties of DPM and its derivatives (Fig. 4c). With two isolated benzene rings, DPM can only emit weak CL (*Φ*_PL_ = 1.4%) within the UV region at 356 nm induced by the weak (π,π) TSC^65^. By introducing one nitrogen atom into the system, *o*-Py-Ph displayed a changed conformation with three modes of TSC, (π,π), (*n*,π), and (σ,π), which strengthen the TSC and largely redshift the wavelength to 534 nm with an increased *Φ*_PL_ of 4.0%. As mentioned before, the pyrimidine-based *o*-2Md with four nitrogen atoms could form a stable planar conformation with strong (*n*,*n*) TSC, boosting its CL wavelength into the NIR region at 700 nm and high efficiency^41^. Fourth, constructing TSC with charge transfer effect (known as through-space charge transfer, TSCT) is an efficient approach to realizing the redshifted CL since the non-conjugated structure can achieve complete charge separation (Fig. 4d)^67–69^. For comparison, the emission properties of the TBC-based DMA-CN-BP and TSCT-based DMA-CN-DPM were measured in different solvents with increased polarity. Their difference is obvious in that the emission color of DMA-CN-BP only shifts from purple to blue, while that of DMA-CN-DPM bathochromically shifts from blue to yellow^40^. Besides, this TSCT can endow non-conjugated CLgens with multiple emission peaks and even single-molecule white-light emission^70^. Focusing on the influence of TSC, the above factors are not all but typical examples to show how to manipulate the photophysical properties of CL, which have been utilized in many polymeric CL materials^71–74^.

In addition, it is worth noting that theoretical calculations play an important role in the development of CL-related theories and materials, especially in the analysis and visualization of transient excited-state processes and TSC of electrons. Virtual screening methods also facilitate the design of CLgens with considerable performance^41^. However, present approaches of theoretical calculation are usually built upon TBC-based theories, showing huge limitations in accurately describing the electronic structure of aggregates and related noncovalent couplings. Thus, theoretical modelings of aggregates, accurate description methods for TSC, as well as machine-learning techniques are urgently needed for the future development and modulation of CL materials.

### Representative potential applications of CL materials

**Fig. 5:**
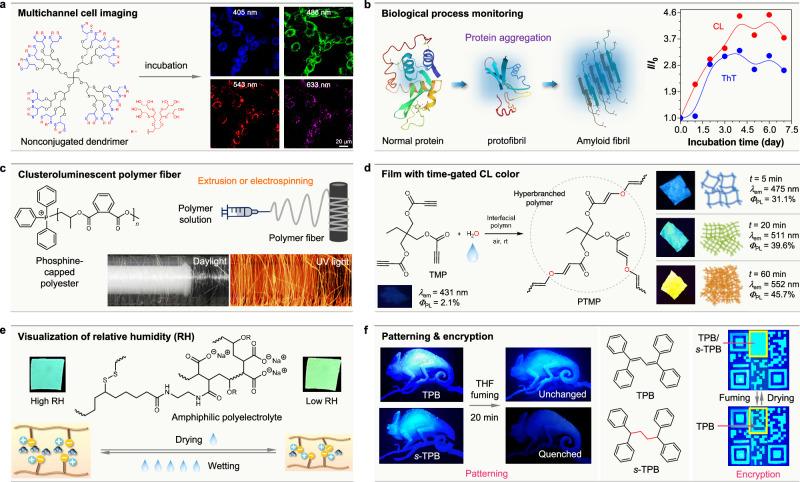
Examples of practical applications of clusteroluminescence (CL). **a** Bioimaging of 4T1 cells by a non-conjugated dendrimer containing oxygen and sulfur atoms; photographs of the cells incubated with the dendrimer taken under photoexcitations at 405, 488, 543, and 633 nm, respectively. **b** The schematic illustration and kinetic curves of the protein-aggregation process of hen egg white lysozyme monitored by its intrinsic CL, data from a commercial dye Thioflavin T (ThT) shown for comparison; *I*_0_ = fluorescence intensity of the native protein, *I* = fluorescence intensity of the aggregated protein. **c** The fabrication of CL fibers from a phosphine-capped polyester and the photographs of the formed fibers taken under daylight and UV light. **d** The synthesis of a hyperbranched polymer (PTMP) by the interfacial polymerization of a non-conjugated triyne monomer (TMP) with water; the chromogen density in the resultant polymer film and its CL color and efficiency readily changed by simply lengthening the polymerization time (*t*). **e** Monitoring humidity change by varying the through-space interaction between water molecules and amphiphilic polymer chains. **f** The difference in the stimuli responsiveness between the TBC-based AIEgen (TPB) and TSC-based CLgen (*s*-TPB); the faster response of the latter to the fuming by tetrahydrofuran (THF) enables 3D fluorescence patterning and 2D information encryption. **a** is adapted with permission from ref. ^76^ Copyright (2021) Springer Nature. **b** is adapted with permission from ref. ^79^ Copyright (2023) The Royal Society of Chemistry. **c** is adapted with permission from ref. ^80^ Copyright (2024) American Chemical Society. **d** is adapted with permission from ref. ^81^ Copyright (2023) Springer Nature. **e** is adapted with permission from ref. ^82^ Copyright (2024) Wiley-VCH Verlag GmbH & Co. **f** is adapted with permission from ref. ^84^ Copyright (2023) American Chemical Society.

With a deeper understanding of the working mechanisms and manipulating strategies of CL, significant efforts have been directed toward exploring potential applications of CL materials. The different structural features, distinct working mechanisms, and varied photophysical properties of CL materials, beyond those of conventional conjugated luminogens, enable specialized applications, as highlighted by several notable examples (Fig. 5). First, with the non-conjugated structures, one crucial feature of CLgens is their high biocompatibility, which promotes them as a different type of bioprobes^75^. For instance, an oxygen/sulfur-based pure *n*-electron dendrimer was demonstrated to produce excitation-dependent emission with a broad excitation window of 450-600 nm, which was successfully utilized for multi-channel bioimaging of 4T1 cells under lasers with different wavelengths (Fig. 5a)^76^. In contrast, it is difficult to achieve multi-channel bioimaging based on traditional conjugated probes. In addition, small-molecule CLgen of diarylmethane was also fabricated as nanoparticles with two-photon CL properties for in vivo angiography of mice, exhibiting high-resolution 3D reconstruction imaging of blood vessels with deep penetration and good biocompatibility^77^. More importantly, with the help of non-covalent interactions during biological aggregation, some natural biomacromolecules were found to play as intrinsic CLgens and achieve visualization of biological processes through their CL signal without external probes^78^. Typically, the aggregation process of hen egg white lysozyme from normal protein to protofibril and amyloid fibril was successfully monitored by its intrinsic CL, which even showed higher sensitivity than the commercial dye of Thioflavin T (ThT) as the external probes (Fig. 5b)^79^.

Second, CL materials with non-conjugated structures are easy to synthesize with simple starting raw materials and show high processability and controllability. For example, using the manual extrusion-spinning method, a phosphine-capped polyester was simply fabricated as large-scale polymer fibers with bright-red CL (Fig. 5c)^80^. Besides, through regulating the time of interfacial polymerization, the non-conjugated triyne-based hyperbranched polymers (PTMP) could be synthesized with controllable CL colors (from blue to yellow) and efficiency (from 31.1% to 45.7%) (Fig. 5d)^81^. These polymeric films based on non-conjugated structure exhibit potential for industrial production with low cost, and their multi-color fluorescence is expected to be utilized for wearable devices, medical sensors, and special safety-warning materials.

Third, since the CL properties are closely related to molecular conformation and non-covalent intra/intermolecular interactions of electrons, CL materials always have sensitive responsiveness to external stimuli, showing changes in CL signal. Following this idea, materials with intrinsic and sensitive stimuli-responsive properties could be achieved. For instance, an amphiphilic polymer containing water-sensitive carboxylate groups was demonstrated to monitor humidity change, which displays redshifted CL from 508 nm to 534 nm with decreased relative humidity from 90% to 10% (Fig. 5e)^82^. Compared to TBC-based luminogens with conjugated structures, TSC-based CLgens exhibit higher sensitivity when responding to the external environment^83^. Thus, two kinds of luminogens with a similar skeleton and the same emissive color (e.g., TPB and *s*-TPB) could be integrated into the same model or code, and utilized for dynamic patterning or information encryption based on their different stimuli responsiveness to solvent fuming (Fig. 5f)^84^. Although most are still at the proof-of-concept stage, the above examples successfully prove CLgens as a different type of luminescent materials, which show the same application potentials as TBC-based luminescent materials and even possess some special features that the latter cannot achieve. Meanwhile, scarce CLgens systems, comparatively poor performance (e.g., short emission wavelength and low efficiency), control of the clustered state, and unknown application scenarios should be further investigated to boost the real-life application potential of CL materials.

### Perspectives from organic clusters and CL

**Fig. 6:**
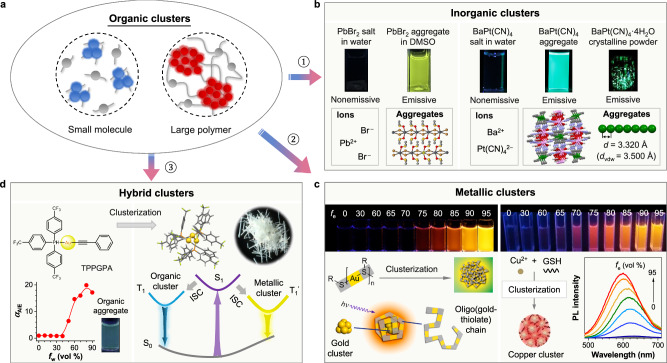
Broaden the CL horizon from organic clusters to inorganic, metallic, and hybrid clusters. **a** The diagrammatic illustration for the CL from the organic clusters of small molecules and large polymers. **b** Examples of CL systems from inorganic clusters: the aqueous solutions of PbBr_2_ and BaPt(CN)_4_ salts are nonemissive as isolated ionic species, but their clusters in the aggregate state are photoluminescent. **c** Examples of metallic CL systems: the aqueous solutions of the oligo(gold-thiolate) chain (left) and the copper salt (right) do not luminesce upon photoexcitation, whose CLs are turned on when large amounts of ethanol (*f*_e_) are added into the water due to the formation of the gold and copper clusters in the aqueous mixtures. **d** An example of CL systems from hybrid clusters: the organic-inorganic hybrid complex (TPPGPA) is nonluminescent in the dilute DMF solution as molecular species, but their organic and metallic clusters in the aggregates emit lights of complementary colors of cyan-blue and yellow, respectively, resulting in the white-light emission of the crystalline aggregates. **b** is adapted with permission from ref. ^87^ Copyright (2021) Wiley-VCH Verlag GmbH & Co. **c** is adapted with permission from ref. ^88^ Copyright (2012) American Chemical Society and ref. ^89^ Copyright (2016) Wiley-VCH Verlag GmbH & Co. **d** is adapted with permission from ref. ^90^ Copyright (2021) Chinese Chemical Society.

Although the emergent CL is mainly observed and investigated in organic systems (e.g., small molecule and large polymer), similar photophysical properties are also found in many inorganic, metallic, and metal-organic hybrid systems that their luminescence properties are mostly correlated with their aggregate characteristics, which could be dated back to 1853^85^. Here, the organic-cluster system could be expanded to three other cluster systems focusing on the fundamental weak interactions and through-space electronic coupling at the aggregate level (Fig. 6a).

For inorganic systems, compounds exist as isolated ionic species in dilute solutions, preventing them from functioning as chromophores to emit visible light. However, the resulting inorganic clusters can serve as luminogens, generating emergent CL in concentrated solution or crystalline powder (Fig. 6b). For example, the isolated ionic species of PbBr_2_ can grow microcrystals with DMSO solvents that change the electronic configuration of Pb^2+^ and stabilize the formed colloids, resulting in green CL originated from PbBr_2_·2DMSO clusters^86^. This similar property is also applied to the inorganic complex of BaPt(CN)_4,_ which is nonemissive in water^87^. However, its aggregates and crystalline clusters with continuous Pt···Pt interactions trigger the through-space *d*-orbitals coupling and electronic delocalization of Pt to form cluster luminogens to emit green CL.

For metallic systems, the emissive species become clusters of metallic cores and are stabilized by the metal-ligand surfaces (Fig. 6c). Xie et al. reported the AIE effect of the oligomeric Au(I)-thiolate complexes, which were not emissive in aqueous solution but generated strong yellow luminescence upon aggregation. Especially, by increasing the thiolate-to-Au ratio, the synthesized Au(0)@Au(I)–thiolate core-shell nanoparticles promote further enhanced intensity of CL from metallic clusters^88^. In addition, Rogach et al. reported the CL properties of all-copper clusters at around 600 nm with the help of glutathione as both the reduction agent and stabilizer^89^.

For metal-organic complexes, they usually possess a CT process between metal and organic ligands, leading to luminescence from a single complex. An interesting gold-based complex, TPPGPA, was designed and synthesized, which exhibits the AIE effect with a cyan-blue color from intramolecular CT upon forming organic aggregates (Fig. 6d). Besides, the crystalline TPPGPA forms a three Au(I)-based hybrid cluster driven by metallophilicity, which results in another emission peak from CL at 578 nm. Thus, organic and metallic clusters together produce the white-light emission of the crystalline TPPGPA aggregates^90^.

From organic clusters to inorganic, metallic, and hybrid clusters, the examples above highlight the crucial role of non-covalent through-space electronic communications across various systems. This enables the emergence of CL from their clusters, which is fundamentally different from their behavior in isolated states. Although current research mainly focuses on organic clusters with CL, the exploration of other systems will also be of great theoretical and experimental value.

**Fig. 7:**
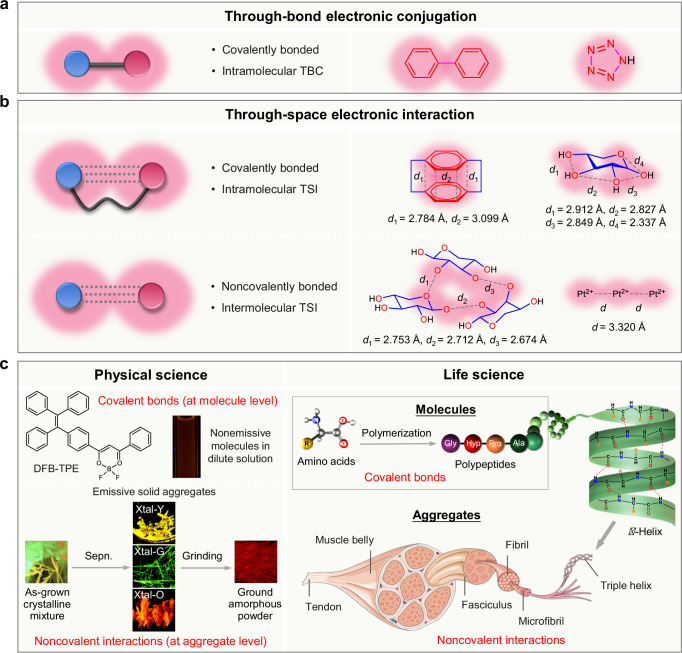
Perspective shift from covalent bonds in molecules to non-covalent interactions in aggregates. **a** When the chromogenic units (e.g., the phenyl rings in biphenyl or the nitrogen atoms in pentazole) are directly linked together, the involved π and lone-pair electrons are delocalized via the TBC (through-bond conjugation). **b** When the chromogenic units (e.g., the phenyl groups in the dibenzenacyclohexaphane ring or the oxygen atoms in the same xylose molecule) are separated by the covalent bonds in between, the electron clouds can be overlapped via the intramolecular TSI (through-space interaction). In non-covalently bonded systems, the chromogenic units (e.g., the oxygen atoms in the different xylose molecules in the monosaccharide aggregate or the platinum ions in the different BaPt(CN)_4_ salts in the inorganic crystal) can experience strong intermolecular TSI (through-space interaction). **c** Examples illustrating the critical importance of non-covalent interactions in physical and life sciences. Left panel: aggregation can turn a non-luminescent molecule (DFB-TPE) luminescent, and different non-covalent interactions can produce different aggregates with varying colors of emission: Xtal-Y, Xtal-G, and Xtal-O (crystals emitting yellow, green, and orange lights, respectively). Right panel: life is a living system of biomolecule aggregates. Above the molecular level, the hierarchical structures of the living aggregates (e.g., *α*-helix, triple helix, microfibril, fibril, fasciculus, muscle belly, and tendon) are mainly determined by the non-covalent interactions at all the structural levels. **c** is adapted with permission from ref. ^94^ Copyright (2020) The Royal Society of Chemistry and the Chinese Chemical Society.

The emergent CL phenomenon and different CL materials discussed above indicate that the TSC of electrons is a typical and general theory for designing and manipulating luminescent materials, which effectively supplements traditional TBC-based photophysical theories. In other words, this special luminescent phenomenon invokes the perspective shift from covalent bonds in molecules to non-covalent interactions in aggregates to better understand and investigate electron interactions and photo-functional materials (Fig. 7). As shown in Fig. 7a, conventional chromogenic units are directly connected to realize electron delocalization and corresponding properties via covalent bonds. TBC-based theories are correct and foundational to some extent, and they focus on the molecular level from a reductionist perspective. However, such theories limit their ability to explain certain photophysical behaviors (e.g., ACQ, AIE, and CL) observed in the aggregate state. On the other hand, through-space interaction (TSI), including TSC and TSCT, provides a different angle from which to review electronic behaviors and material properties. Here, two general models with TSI are proposed (Fig. 7b): (1) The chromogenic units are indirectly connected by saturated covalent bonds with intramolecular TSI, as illustrated by dibenzenacyclohexaphane and xylose with intramolecular (π,π) and (*n*,*n*) interactions, respectively. This model still stands at the molecular level but shows electronic processes beyond covalent bonds. (2) The chromogenic units are separated and spatially connected by intermolecular interactions, as illustrated by xylose and Pt(CN)_4_ clusters. This model only exists in the aggregate state to facilitate non-covalent and intermolecular electronic communications.

Actually, the material world and living organisms are built based on multiple non-covalent interactions at the aggregate level, demonstrating their importance in physical and life sciences^8,91,92^. In physical science, different states and non-covalent interaction modes can cause covalently bonded molecules to show different characteristics at different structural hierarchies^93^. For example, the luminescence of DFB-TPE is turned on by the aggregation process, where multiple intermolecular non-covalent interactions restrict intramolecular motions. More importantly, different packing and networks of non-covalent interactions in the crystalline state endow DFB-TPE to emit yellow, green, and orange lights, respectively (Fig. 7c, left panel)^94^. Apart from luminescence, non-covalent interactions could also modulate other physicochemical properties of materials, such as the morphology of aliphatic polymers functionalized with amide/imide groups and the electrical performance of alkoxy-functionalized bithiophene/thiazoles as organic semiconductors^95^. In addition, living organisms rely more on multiple non-covalent interactions in the aggregate structure. For instance, while amino acids are covalently bonded to form polypeptides, the macroscopic structures and functions of *α*-helices, microfibrils, fasciculi, muscle bellies, and tendons rely on complex non-covalent interactions within these biomolecular aggregates (Fig. 7c, right panel)^96^. Starting from nature to understand the features and interactions of matters, non-covalent interactions should be placed equally or even higher than traditional covalent interactions. This also puts higher demands on researchers to think about physical and life sciences from a macroscopic and holistic perspective^97^.

### Summary and outlook

Motivated by the widely existing phenomenon and special photophysical behaviors, CL and related materials have witnessed a quick development in the past several years. As an emerging type of luminescent materials, CLgens show uncommon properties, such as non-conjugated structures, clustered emitting species, unmatched absorption and excitation spectra, excitation-dependent emission, and RTP phenomenon. Based on reported examples, small-molecule CLgens have provided some typical models to illustrate structure-property relationships and guided the design of polymeric systems with better photophysical performance. From the working mechanism of CL, the TSC endows non-conjugated compounds with a strong conjugation effect of electrons to achieve luminescence within the visible-light and NIR regions, which also supplements the traditional TBC-based photophysical theories. Many factors affecting TSC and CL properties are investigated, including dihedral angle, electron density, heteroatoms with lone pairs, and through-space CT effect, which could be utilized for designing and modulating CL materials. Due to their different features, CLgens are poised to have tremendous potential applications, such as multi-channel bioimaging, biological process monitoring, multi-color luminescent fibers, environmental humidity sensing, and information encryption. The above aspects discussed in this perspective may provide a comprehensive picture of CL research at the current stage, invoking more insightful consideration and development vitality into this booming field.

Although the working mechanism of CL has become clearer and some CLgens with excellent performance have been developed, the emergent CL is still in its infancy. Thus, increasing explorations are necessary to uncover all aspects of CL with some important views: (1) deeper elucidations of electronic TSC based on non-covalent interactions at the aggregate state, especially for the theoretical modeling of electron coupling of aggregates^98,99^; (2) more efficient and feasible strategies for regulating properties of CLgens since the performance of most CLgens still lags behind that of traditionally conjugated luminescent materials^62^; (3) expanded CL systems from natural to synthetic materials, from small molecules to polymers, and from organic clusters to inorganic/metallic/hybrid clusters; (4) further exploitations of potential applications of CLgens, especially for biological probes and phototheranostics due to their high biocompatibility and biodegradability; (5) understanding non-covalent interactions in biological systems, particularly for their crucial roles in protein aggregation or biomolecular self-assembly as intrinsic CLgens^100^.

As inspired by physical and life sciences, non-covalent interactions should play an equal and even priority role to traditional covalent interactions in determining properties and functions of materials, especially at high levels of structural hierarchy. Thus, the perspective shift from covalent bonds at the molecular level to non-covalent interactions at the aggregate level is necessary for CL research and other aspects of materials science. The development of CL is anticipated to emerge as a distinct research field of aggregate science, paving the way for a different category of photophysics and photo-functional materials.

## Methods

### Materials

Xylitol, diethyl ether, and lead(II) bromide were purchased from Energy Chemical, poly(ethylene glycol) (average *M*_n_ = 600) was purchased from Shanghai Macklin Biochemical Technology Co., Ltd., poly(ethylene glycol) (average *M*_n_ = 8000) was purchased from Aladdin Scientific Corp., starch was purchased from Shanghai Lingfeng Chemical Reagent Co., Ltd., and bovine serum albumin was purchased from MP Biomedicals, LLC.
